# Risk of symptomatic COVID‐19 due to aircraft transmission: a retrospective cohort study of contact‐traced flights during England’s containment phase

**DOI:** 10.1111/irv.12846

**Published:** 2021-03-01

**Authors:** Paula Bianca Blomquist, Hikaru Bolt, Simon Packer, Ulf Schaefer, Steven Platt, Gavin Dabrera, Maya Gobin, Isabel Oliver

**Affiliations:** ^1^ UK Field Epidemiology Training Programme Public Health England London United Kingdom; ^2^ North West Field Services National Infection Service Public Health England Liverpool United Kingdom; ^3^ South East and London Field Services National Infection Service Public Health England London United Kingdom; ^4^ South West Field Services National Infection Service Public Health England Bristol United Kingdom; ^5^ Core Bioinformatics Group, Data and Analytical Sciences National Infection Service Public Health England London United Kingdom; ^6^ National Infection Service Public Health England London United Kingdom

**Keywords:** Aircraft, Contact tracing, COVID‐19, Epidemiology, SARS‐CoV‐2, Transmission

## Abstract

**Background:**

Knowledge gaps remain regarding SARS‐CoV‐2 transmission on flights. We conducted a retrospective cohort study to estimate risk of acquiring symptomatic SARS‐CoV‐2 on aircraft, to inform contact tracing and infection control efforts.

**Methods:**

We identified co‐passengers of infectious passengers on 18 England‐bound flights from European cities up to 12/03/2020, using manifests received for contact tracing. Infectious passengers were laboratory‐confirmed cases with symptom onset from 7 days before to 2 days after the flight. Possible aircraft‐acquired cases were laboratory‐confirmed with onset 3‐14 days post‐flight with no known non‐flight exposure. Manifests was merged with the national case management dataset (identifying cases, onset dates, contact tracing status) and the national COVID‐19 linelist. Contact tracing notes were reviewed to identify non‐flight exposures. We calculated attack rates (ARs) among all co‐passengers and within subgroups, including by distance from infectious cases and number of infectious cases on‐board.

**Results:**

There were 55 infectious passengers and 2313 co‐passengers, including 2221 flight‐only contacts. Five possible aircraft‐acquired cases were identified; ARs of 0.2% (95%CI 0.1‐0.5) among all flight‐only contacts and 3.8% (95%CI 1.3‐10.6) among contact‐traced flight‐only contacts sat within a two‐seat radius. The AR among 92 co‐travellers with known non‐flight exposure to infectious cases was 13.0% (95%CI 7.6%‐21.4%). There were insufficient numbers to assess differences between subgroups.

**Conclusion:**

We conclude that risk of symptomatic COVID‐19 due to transmission on short to medium‐haul flights is low, and recommend prioritising contact‐tracing of close contacts and co‐travellers where resources are limited. Further research on risk on aircraft is encouraged.

## 1 BACKGROUND

The COVID‐19 pandemic is a global health emergency,^1^ with more than 100 million cases reported worldwide by the end of January 2021.^2^ During the containment phase in England (up to 12th March 2020),^3^ Public Health England (PHE) implemented control measures to prevent case importation. In addition to supported isolation of travellers from high‐incidence areas, PHE followed an air travel contact‐tracing protocol similar to that of other respiratory infections, which involved identifying cases with recent flight history and liaising with port health authorities and airline companies to identify their contacts.^4^ Where reachable, contacts were put under passive surveillance for 14 days from the day of the flight, and, if they developed symptoms, asked to inform PHE and call NHS 111 to get tested. In doing this, PHE defined an air travel contact as a person sitting within a two seat radius of a confirmed case, or a member of cabin crew serving the area around where the case was seated.^4^

The magnitude and determinants of risk of SARS‐CoV‐2 transmission on aircraft continue to be of public and policy interest. The international aviation industry assesses risk to be low,^5^ pointing to high‐efficiency particulate air (HEPA) filters installed on aircraft, vertical air flow, and seats acting as physical barriers. Published studies have varied in their risk assessment. There were no secondary cases identified among 350 co‐passengers of an infectious case on a 15 hours flight between Wuhan China and Toronto Canada,^6^ or among 326 co‐passengers monitored in the Northern Territory of Australia.^7^ However a case who flew from Central African Republic to France in February 2020 was identified as possibly aircraft‐acquired,^8^ whole‐genome sequencing has suggested direct transmission between two passengers and two crew members on a 15 hour flight from Boston to Hong Kong,^9^ and there were 16 possible secondary cases identified after exposure on a 10 hours commercial flight from London to Vietnam – an attack rate of 62% in the business class section.^10^ A review of SARS case reports of in‐flight transmission during the 2003 pandemic found two flights where half of secondary SARS cases were sat beyond the two‐row limit, which equated to a total 26% attack rate among passengers sat within two rows of the infectious individual and 7% among passengers sat beyond this.^11^ A Lancet meta‐analysis determined that infection risk due to physical contact with a contagious person was 13%, and this notably decreased with additional each metre of distance.^12^

This study aimed to estimate the risk and modifiable risk factors of symptomatic COVID‐19 due to transmission on aircraft, to inform contact‐tracing methods, infection prevention and control efforts, as well as travel‐related policy and guidance more widely.

## 2 METHODS

### 2.1 Study setting and period

This was a retrospective cohort study based on contact‐traced international aircrafts that flew to England during the containment period, which occurred between early January and March 12th 2020. Only flights with an available manifest and a known seat number of any presumed infectious cases were included.

### 2.2 Case definitions

Index cases were passengers with a laboratory‐confirmed SARS‐CoV‐2 test with symptom onset between seven days before or up to two days after an international flight.

Cases among flight contacts with symptom onset (or specimen date if symptom dates were missing) between 3 and 14 days after the flight were considered “additional cases.” These were subcategorised as follows:

- **Possible aircraft‐acquired case**: confirmed case among co‐passengers whose only known exposure to the case was on the flight and had no whole‐genome sequencing evidence to rule out direct transmission.
- **Multiple‐exposure case**: confirmed case among co‐passengers with evidence of household or other travel contact with an index case, as indicated in contact‐tracing records or by having the same traveller information in the flight manifest as an index case (same personal contact email, record locator code, or booking reference number in the manifest).

All cases were presumed to be symptomatic, even where onset dates were missing, due to testing criteria at the time. Multiple‐exposure cases were considered more likely to acquire infection from an index case during close contact outside the flight. We assume that passengers were not wearing masks during the flight due to this being in the early stages of the pandemic before mask wearing was widespread.

### 2.3 Data sources

We accessed and compiled data from the following sources:

#### 2.3.1 Airline manifests

Flight datasets shared by airlines with PHE, which contained passenger names, demographics, home address, booking email addresses, booking reference numbers, and seat numbers.

#### 2.3.2 HPZone

The case management system used by all local PHE Health Protection Teams to record cases, contacts, and communications related to the case management or contact tracing. The data extracted included details of COVID‐19 test results, onset dates, and contextual information such as contacts outside of flights or movement between seats.

#### 2.3.3 PHE line list of COVID‐19 cases

A national line list of laboratory‐confirmed cases in England reported to PHE, including specimen dates, names, and demographic details of confirmed cases.

#### 2.3.4 Contact‐tracing logs and communications

Documentation created locally to facilitate the contact‐tracing process, including logs of case seat numbers and emails exchanged between PHE staff to initiate and report progress on contact tracing.

#### 2.3.5 FlightAware.com

An online database of past international flights, including actual recorded length of flight and aircraft model.

#### 2.3.6 COG‐UK data

Database of SARS‐CoV‐2 sequences sequenced in England (data release COG20200601), maintained by the COVID‐19 Genomics UK consortium.

### 2.4 Data collection and management

All available manifests were standardised and appended, then restricted to passengers who had boarded the flight. The full manifest dataset was linked to HPZone data on name as well as date of birth and/or residential postcode to identify cases, onset dates, and persons who were contact traced. Contact‐tracing logs and communications were manually reviewed to fill in information gaps and identify persons with multiple‐exposures, and FlightAware.com was queried for flight length per flight.

To capture further cases, the manifest dataset was then linked with the England COVID‐19 laboratory line list. Line list cases were considered matches if they matched the manifest record exactly on last name and date of birth/postcode, or, if the manifest did not include date of birth/postcode but they matched exactly on name alone and there was evidence of foreign travel to the country in question in HPZone records.

Microsoft Excel was used for data management where manual compilation of information was required. R version 3.5.3 was used for manipulation of datasets, including labelling of cases and exposure categories.

### 2.5 Whole‐genome sequencing

COG‐UK data were searched to identify pre‐aligned sequences from pairs or groups of index and possible aircraft‐acquired cases who shared a flight. Sequences from multiple‐exposure cases from these flights or from other flights for which only sequences from index or only possible aircraft‐acquired cases were available were selected as background sequences. Pangolin software was used to calculate global lineages, and a phylogenetic tree generated with iqtree2. Pairwise distances between sequences were further calculated using SNP‐distances.

### 2.6 Analysis

The number of additional cases with onset 3 to 14 days after the flight was presented overall and by onset date. Attack rates were calculated among all co‐passengers and among those who were successfully contacted to inform them of the contact‐tracing and surveillance procedures (referred to as “contact traced” hereafter). Possible aircraft‐acquired cases were identified, taking into account WGS evidence where available, and risk of aircraft transmission was estimated among co‐passengers whose only known exposure to the index case was on the flight. This was compared to attack rates among multiple‐exposure contacts of index cases. Possible aircraft‐acquired cases were described in terms of time, person, and place on the aircraft. Finally, a risk factor analysis was undertaken by presenting attack rates by distance from the index case, number of index cases on the flight or in close proximity, flight length, and city of departure.

### 2.7 Ethical considerations

Data were collected for contact‐tracing and health protection purposes, falling under Regulation 3 of the Health Service (Control of Patient Information) Regulations 2002. Regulation 3 specifically relates to communicable disease and other risks to public health and as such encompasses contacting activities.

## 3 RESULTS

### 3.1 Study population

Contact tracing was initiated for 45 flights that landed in England between 31 January and 12 March 2020. Full manifests with unique seat numbers, including at least one seat specified for an index case, were available for 18 flights. These flights departed from across Europe (Table 1), with a median flight time of 115 minutes (range 86 to 259 minutes). 2368 passengers were recorded to have boarded these flights, of whom 2213 had an available date of birth or postcode to allow matching with the COVID‐19 laboratory line list (93.5%). Among those with available sex (n = 770) or age (n = 1009), 64% (n = 496) were male, and the median age was 41 years (interquartile range 28 to 53).

**TABLE 1:** Number of flights, passengers, and COVID‐19 index cases on England‐bound flights between 31 January 2020 and 12 March 2020 contact traced by PHE for which manifests were available for analysis

Location of departure	Flights	Passengers	Index cases	Index cases per 100 passengers
Austria (Innsbruck)	5	758	20	2.6
Germany (Berlin)	1	158	2	1.3
Italy	8	993	22	2.2
Milan	3	227	4	1.8
Turin	1	130	2	1.5
Verona	4	636	16	2.5
Spain (Tenerife)	1	181	1	0.6
Switzerland	2	129	8	6.2
Basel	1	103	1	1.0
Geneva^*^	1	26	7	26.9
Turkey (Istanbul)	1	149	2	1.3
Total	18	2368	55	2.3

* Incomplete manifest for one flight

Fifty‐five persons meeting the case definition of an index case were identified, including one person who tested positive in another country. No further index cases were identified through linkage with the national line list, although linkage verified all 54 index cases tested in England. Forty index cases were symptomatic on the flight, and 15 developed symptoms in the two days after the flight.

Of the 2313 co‐passengers, 2221 were flight‐only contacts and 92 were multiple‐exposure contacts. Of the former group, 425 sat within two seats of an index case, thereby meeting the definition of an individual who should be contact traced based on the flight contact alone. Of these, 79 (19%) persons were recorded in HPZone as successfully contact traced.

### 3.2 Additional cases and attack rates

**FIGURE 1:**
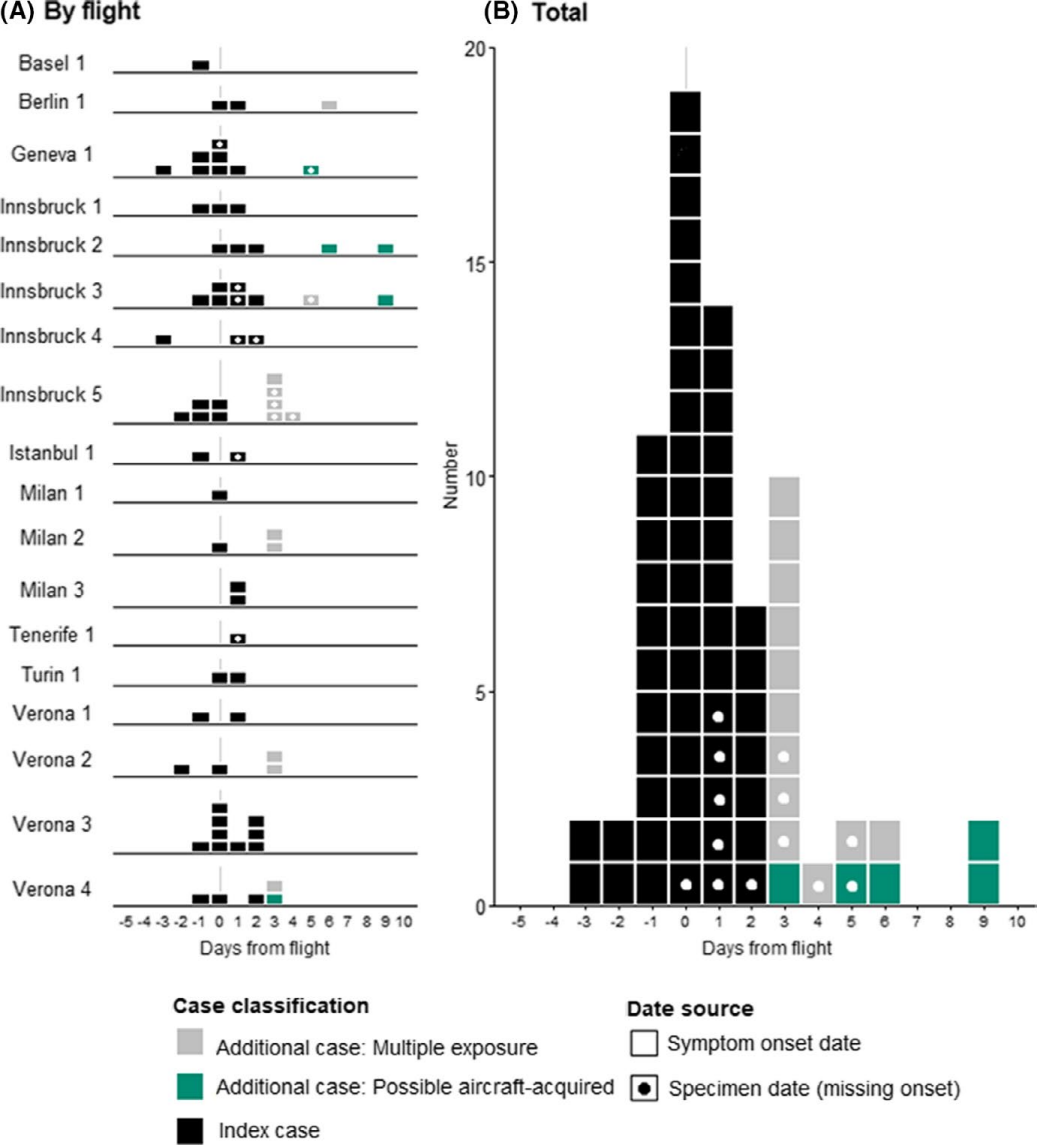
Epicurve of days from flight to onset date/specimen date in COVID‐19 cases on analysed England‐bound contact‐traced flights between 31 January 2020 and 12 March 2020, by case classification. Flight pseudonyms used

Among all 2,313 co‐passengers, there were 17 COVID‐19 cases with symptom onset or specimen date 3 to 14 days after the flight (Figure 1; Table 2). Five of these cases were possible aircraft acquired, among the 2,221 persons with flight‐only contact: an attack rate of 0.2% (95% CI 0.1‐0.5). Restricting to those sat within two rows and contact traced, the attack rate increased to 3.8% (95% CI 1.3‐10.6).

**TABLE 2:** Number and proportion of additional COVID‐19 cases among co‐passengers of index cases on analysed England‐bound contact‐traced flights between 31 January 2020 and 12 March 2020

	Total				Sat within two seats			
	N	Cases	AR (%)	[95%CI]	N	Cases	AR (%)	[95%CI]
All co‐passengers								
Total	2313	17	0.7	[0.5‐1.2]	480	10	2.1	[1.1‐3.8]
Contact traced	102	15	14.7	[9.1‐22.9]	91	9	9.9	[5.3‐17.7]
Flight‐only contacts								
Total	2221	5	0.2	[0.1‐0.5]	425	4	0.9	[0.4‐2.4]
Contact traced	79	3	3.8	[1.3‐10.6]	79	3	3.8	[1.3‐10.6]
Multiple‐exposure contacts								
Total	92	12	13.0	[7.6‐21.4]	55	6	10.9	[5.1‐21.8]
Contact traced	23	12	52.2	[33.0‐70.8]	12	6	50.0	[25.4‐74.6]

More than two thirds of additional cases (n = 12) were among multiple‐exposure contacts, with evidence of close contact with the index case outside the flight: an attack rate of 13.0% (7.6‐21.4). Among close contacts who were followed up, one in two developed symptomatic COVID‐19.

### 3.3 Whole‐genome sequencing

A total of 107 sequences on nine flights were identified. No flights were identified on which sequences were available from both the index case and any of five of the possible aircraft‐acquired cases, so this information could not be used to support or rule out direct transmission.

### 3.4 Description of possible aircraft‐acquired cases

**FIGURE 2:**
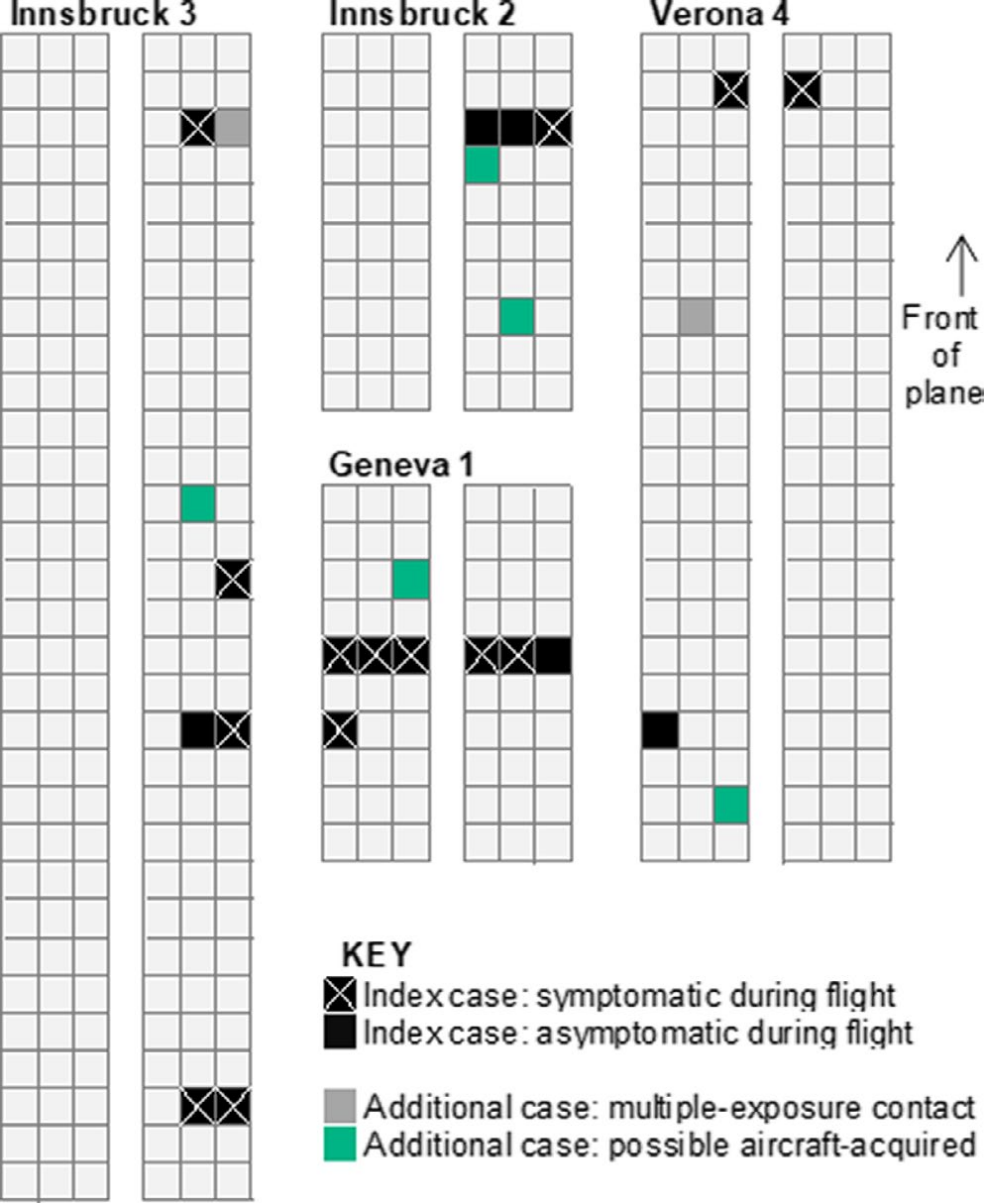
Seating arrangement of COVID‐19 cases on flights with possible aircraft transmission of SARS‐CoV‐2, among analysed England‐bound contact‐traced flights between 31 January 2020 and 12 March 2020, by case classification. Flight pseudonyms used. Seating area limited to area around cases and seats of other passengers not indicated

The five possible aircraft‐acquired cases were four males and one female ranging between 32 and 60 years of age, who travelled on four flights from Innsbruck, Geneva, and Milan. Their flight times ranged from 109 to 120 minutes. These persons were sat throughout the plane body, and at varying distances from the closest index case(s) (Figure 2): four were sat within two seats, and one was sat five rows away. Each of these flights had at least three index cases and at least one symptomatic index case.

### 3.5 Risk factor analysis

Only one case was detected beyond the two‐seat radius eligible for contact tracing and no meaningful difference in crude risk was found comparing to those sat within two seats (Table 3). There were no possible aircraft‐acquired symptomatic cases among the 19 persons sat directly next to an index case, but only two of these persons were successfully contact‐traced. Among contact‐traced individuals, the attack rate was higher if sat within two seats of two index cases (6.2%) than if sat within two seats of one index case (2.1%), or within two seats of a symptomatic case compared to an asymptomatic case (3.9% vs 0.0%), however with notably overlapping confidence intervals.

**TABLE 3:** Secondary attack rates among co‐passengers with no close contact with index case(s) on analysed England‐bound contact‐traced flights between 31 January 2020 and 12 March 2020

	Eligible flight contacts			Eligible flight contacts with initiated contact‐tracing		
	N	Cases	AR (%) [95% CI]	N	Cases	AR (%) [95% CI]
Individual‐level factors						
Distance from closest index case						
Beyond 3 rows	1465	1	0.1 [0‐0.4]	0	0	
3 seats away	331	0	0.0 [0‐1.1]	0	0	
2 seats away	270	3	1.1 [0.4‐3.2]	50	2	4.0 [1.1‐13.5]
1 seat away on different row	132	1	0.8 [0.1‐4.2]	27	1	3.7 [0.7‐18.3]
1 seat away on same row	23	0	0.0 [0.0‐14.2]	2	0	0.0 [0.0‐65.8]
Number of index cases within 2 seats						
0	1796	1	0.1 [0.0‐0.3]	0	0	
1	248	2	0.8 [0.2‐2.9]	47	1	2.1 [0.4‐11.1]
2	177	2	1.1 [0.3‐4.0]	32	2	6.2 [1.7‐20.1]
Symptoms of closest index case, among those with index case within 2 seats						
Asymptomatic	90	1	1.1 [0.2‐6.0]	3	0	0.0 [0.0‐56.1]
Symptomatic	335	3	0.9 [0.3‐2.6]	76	3	3.9 [1.4‐11.0]
Flight‐level factors						
Number of index cases on flight						
1 (4 flights)	458	0	0.0 [0.0‐0.8]	17	0	0.0 [0.0‐18.4]
2 to 4 (10 flights)	1272	3	0.2 [0.1‐0.7]	34	1	2.9 [0.5‐14.9]
5+ (4 flights)	491	2	0.4 [0.1‐1.5]	28	2	7.1 [2.0‐22.6]
Length of flight						
< 1.5 hours (1 flight)	143	0	0.0 [0.0‐2.6]	0	0	
1.5 to 2.5 hours (15 flights)	1754	5	0.3 [0.1‐0.7]	58	3	5.2 [1.8‐14.1]
4 to 5 hours (2 flights)	324	0	0.0 [0.0‐1.2]	21	0	0.0 [0.0‐15.5]
Country of departure						
Austria	724	3	0.4 [0.1‐1.2]	35	2	5.7 [1.6‐18.6]
Germany	155	0	0.0 [0.0‐2.4]	3	0	0.0 [0.0‐56.1]
Italy	898	1	0.1 [0.0‐0.6]	11	0	0.0 [0.0‐25.9]
Spain	177	0	0.0 [0.0‐2.1]	11	0	0.0 [0.0‐25.9]
Switzerland	120	1	0.8 [0.1‐4.6]	9	1	11.1 [2.0‐43.5]
Turkey	147	0	0.0 [0.0‐2.5]	10	0	0.0 [0.0‐27.8]
Total (18 flights)	2221	5	0.2 [0.1‐0.5]	79	3	3.8 [1.3‐10.6]

Similarly, attack rates were higher in flights with more index cases, with no additional cases in the four flights with only one index case, three in the ten flights with two to four index cases (0.2% attack rate), and two in the four flights with four or more index cases (0.4%). There were insufficient numbers or variation in flight length to observe patterns by flight length.

## 4 DISCUSSION

In this study of flight, contact‐tracing, and laboratory data of passengers on 18 England‐bound aircraft, we identified 55 COVID‐19 index cases presumed to be infectious during travel. There were 17 further symptomatic COVID‐19 cases with symptom onset or specimen dates within 3 to 14 days after the flight. Only five of these individuals were possible aircraft‐acquired cases, representing an attack rate of 0.2% among those whose only known encounter with the index case was on the flight, and 3.8% when further restricting to those who were contact‐traced. Due to smaller numbers of those contact‐traced, the latter estimate ranged from 1.3% to 10.5% (based on the attack rate confidence interval). These risks were considerably lower than that of multiple‐exposure contacts of the index case: 13.0% overall (95% CI 7.6 to 21.4%) and 52.2% (95%CI 22.0 to 70.8%) among those contact‐traced.

The five possible aircraft‐acquired cases were sat on four of the 18 flights studied, travelling from Innsbruck, Geneva, and Milan, and were sat between one row and five rows away from the closest index case. Due to small numbers, there was insufficient evidence of the relationship between risk of SARS‐CoV‐2 acquisition and seat proximity, number of index cases on the flight or nearby, or whether a nearby index case was experiencing symptoms. Differences in case ascertainment prevent comparisons between those sat within and beyond a radius of two seats from the index case, as the former were eligible for close symptom monitoring and testing via contact tracing. There were no cases among those sat directly adjacent to a case, but only two persons sat adjacent to a case and were contact‐traced. We note that several persons who sat adjacent to an index case were close contacts that were excluded from the risk factor analysis. Additionally, we could not assess the impact of flight time on risk, as all cases were on flights between 1.5 and 2.5 hours long, and not on shorter or longer flights.

This study benefits from a wealth of data collected for COVID‐19 contact‐tracing purposes, as well as a thorough method using both manual review and data linkage. In particular, this included the extraction of onset dates (found in 59 of 72 cases), and identification of passengers who lived or travelled with an index case, by reviewing flight booking information in the manifest as well as contact‐tracing notes. Due to linkage to the national line list using electronic airplane manifests, which typically contain accurate patient information, we are reasonably confident that we identified most, if not all, laboratory‐confirmed cases associated with these flights.

However, risk of transmission in this setting remains difficult to quantify, and under‐ascertainment is a major limitation to this study. Our risk estimate does not represent total risk of acquiring infection, including asymptomatic cases, which could only be estimated if all passengers were systematically tested. It is likely that there were several undetected cases, including mildly symptomatic cases or persons not reached by contact tracing, who therefore did not know they were eligible for testing.^13^ Other cases may have been unknown to PHE due to onward travel and diagnosis abroad.

Misclassification of exposure may also have occurred: some “flight‐only” cases or seat numbers may have been misidentified due to incomplete information on passenger interactions or movements. The majority of passengers who sat within two rows of an index case were not contact‐traced, in part because contact tracing for some flights was ceased as England transitioned from containment to delay phase, and because contemporaneous guidance only considered a passenger to be infectious if symptomatic on the flight.^4^

On balance, it is likely that we have underestimated the number of passengers who were infected with SARS‐CoV‐2, but among them we may have overestimated the number that were due to acquisition on aircraft. Acquisition of SARS‐CoV‐2 may have occurred prior to departure, at other points during travel, or upon return.^14^ Pre‐departure acquisition is particularly likely due to the high incidence in the departures cities at the time,^15^ and further indicated by the short time difference between flight and symptom onset for some of the cases, in particular the secondary case with symptoms on the third day after the flight on the Verona 4 flight.

We conclude that risk of symptomatic COVID‐19 due to transmission on short to medium haul flights is likely low, at approximately 3% but less than 10% if sat within two rows of an infectious individual. This is consistent with the low numbers of aircraft‐acquired secondary cases seen in some other published contact‐tracing analyses,^6^, ^7^, ^8^ although lower the 62% attack rate found in Khanh et al's^10^ study of a 10 hours flight. These differences may be because we investigated shorter flights, ruled out more close contacts from possible aircraft transmission, or had lower case ascertainment. Differences in aircraft ventilation and filtering systems are also possible,^16^ although these details were unavailable for this study.

The risk identified in this paper is relevant to England's containment period, so may be higher than risk on flights later in the pandemic, due to measures taken within the aviation industry. Guidance developed by the European Union Aviation Safety Agency and the European Centre for Disease Prevention and Control in spring 2020 ^16^ recommended that aircraft operators minimise in‐flight service, install HEPA filters if not already in place, regularly disinfect aircraft, and encourage use of medical face masks and adherence to respiratory etiquette and hand hygiene among passengers. Mask wearing on flights has since been mandated on many airlines (examples in references ^17^, ^18^, ^19^). Additionally, pre‐departure negative COVID‐19 PCR test results and “Fit‐to‐fly” certificates are required by several receiving countries,^20^, ^21^, ^22^ with such testing becoming more readily available.^23^ True risk of both encountering and acquiring SARS‐CoV‐2 from an infectious co‐traveller may consequently be lower, although sensitivity of detecting in‐flight transmission events may be improving in line with guidance for aircraft operators to ensure thorough record keeping of passenger information to facilitate contact tracing.^16^

This study was not able to propose specific social distancing recommendations on aircraft or changes to the two‐seat eligibility criteria for contact tracing. While cases beyond two rows were identified, we also cannot rule out transmission outside of the flight for these persons. However, the significantly higher attack rates among close contacts and co‐travellers suggest these should be prioritised over persons with flight‐only contact where resources are limited, and particularly where flight journeys are short. Such contacts are likely higher risk due to closer and more prolonged exposure, unlike unfamiliar co‐passengers who are less likely to interact and face each other during the flight.^5^ With potential for asymptomatic infection and more than a quarter of index cases developing symptoms after the flight, our findings also highlight the importance of post‐flight quarantine from high‐incidence areas to prevent case importation. There should be continued efforts to study and communicate modifiable risk factors for transmission on flights, to inform flight planning and to help travellers make informed choices on travel abroad.

## CONFLICT OF INTEREST

We have no conflicts of interest to declare.

## AUTHOR CONTRIBUTION

**Paula B Blomquist:** Data curation (lead); Formal analysis (lead); Investigation (lead); Methodology (lead); Project administration (lead); Software (lead); Writing‐original draft (lead); Writing‐review & editing (lead). **Hikaru Bolt:** Investigation (supporting); Methodology (supporting); Software (supporting); Validation (supporting); Writing‐review & editing (supporting). **Simon Packer:** Investigation (supporting); Writing‐review & editing (supporting). **Ulf Schaefer:** Data curation (supporting); Investigation (supporting); Software (supporting). **Steven Platt:** Data curation (supporting); Investigation (supporting). **Gavin Dabrera:** Data curation (supporting); Project administration (supporting). **Maya Gobin:** Conceptualization (equal); Writing‐review & editing (supporting). **Isabel Oliver:** Conceptualization (equal); Supervision (equal); Writing‐review & editing (supporting).

### PEER REVIEW

The peer review history for this article is available at https://publons.com/publon/10.1111/irv.12846.
